# Supplementary material for: KAP1 regulates endogenous retroviruses in adult human cells and contributes to innate immune control
Source: EMBO Rep. 2018 Jul 30;19(10):e45000. doi: 10.15252/embr.201745000 (PMC6172469; doi:10.15252/embr.201745000)
Supplement: Supplementary file 1 — Appendix [file EMBR-19-e45000-s001.pdf]

Appendix

KAP1 regulates ERVs in adult human cells and contributes to innate immune control

Christopher H.C. Tie[1], Liane Fernandes[1], Lucia Conde[2], Luisa Robbez-Masson[1], Rebecca P. Sumner[1], Tom Peacock[1], Maria Teresa Rodriguez-Plata[1], Greta Mickute[1], Robert Gifford[3], Greg J. Towers[1], Javier Herrero[2] and Helen M. Rowe[1]

[1]Division of Infection and Immunity, Cruciform Building, University College London, WC1E 6BT, UK
[2]Bill Lyons Informatics Centre, UCL Cancer Institute, Paul O'Gorman Building, 72 Huntley Street, London WC1E 6BT, UK
[3]MRC-University of Glasgow Centre for Virus Research, G61 1QH, UK
Corresponding author: h.rowe@ucl.ac.uk

Appendix Table of Contents

Appendix Figure S1	Page 2
Appendix Figure S2	Page 3
Appendix Table S1	Page 4
Appendix Table S2	Page 5-6
Appendix Table S3	Page 7
Appendix Figure S1 Legend	Page 8
Appendix Figure S2 Legend	Page 9

Appendix Figure S1

A.

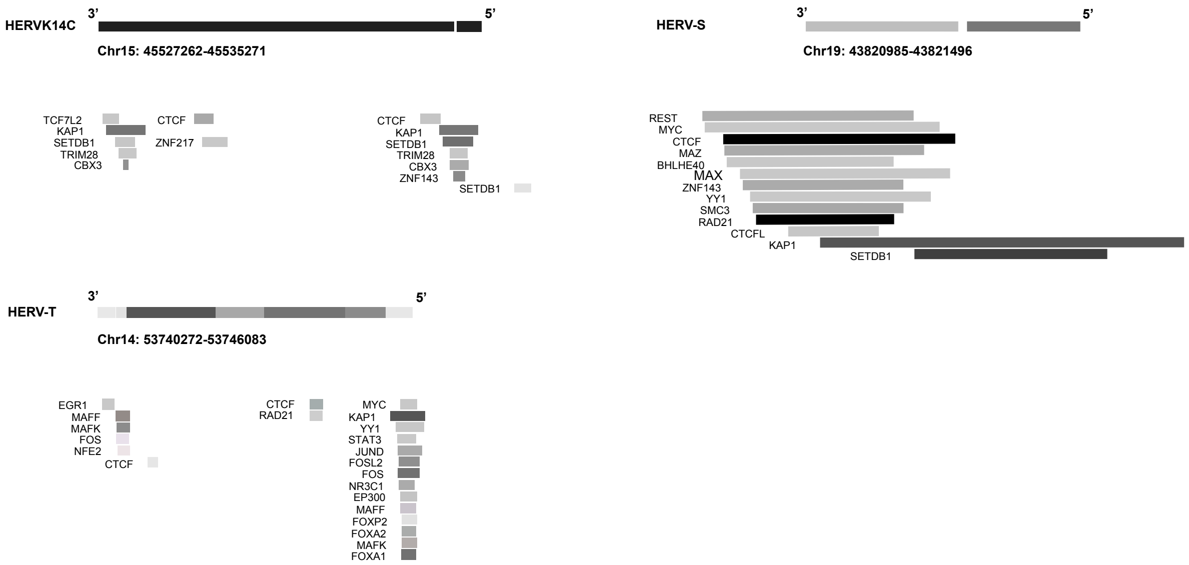

B.

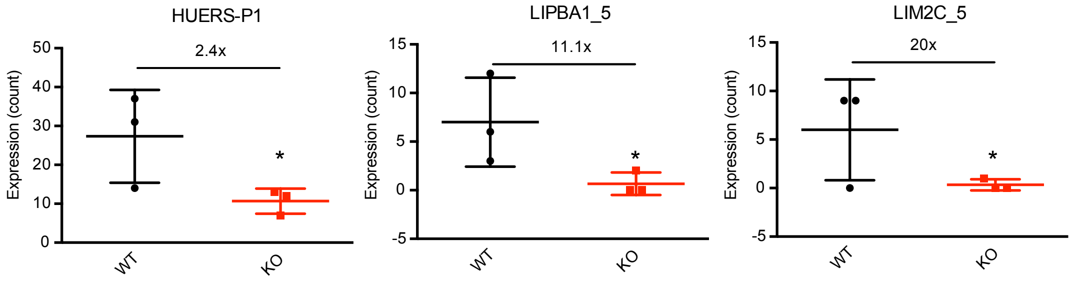

Appendix Figure S2

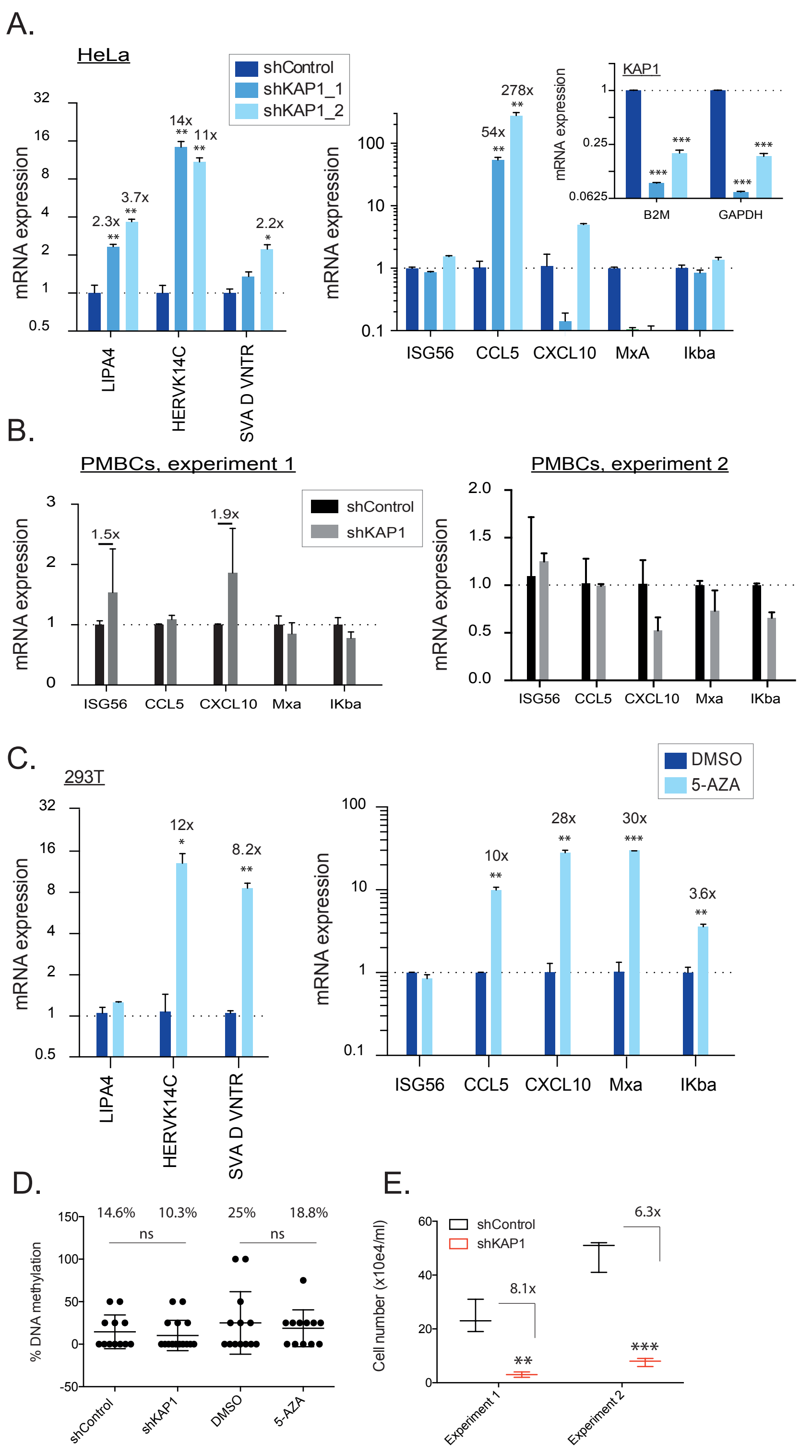

Appendix Table S1

Antibodies

Actin	Chemicon, MAB1501	mouse monoclonal
KAP1	Millipore, MAB3662	mouse monoclonal
PCNA	Millipore, clone PC10	mouse monoclonal
OCT3/4	Santa Cruz, sc-5279	mouse monoclonal
PE-Cγ 5 CD3 (ε-chain)	BD Bioscience, 555341	mouse
FITC - CD4	BD Bioscience, 345768	mouse
PE - OCT3/4	eBioscience, 12-5841-80	rat
FITC IgG1 (κ Isotype Control)	BD Bioscience, 555909	mouse
PE-Cγ 5 IgG2a (κ Isotype Control)	BD Bioscience, 555575	mouse
PE IgG2a (κ Isotype Control)	eBioscience, 12-4321-80	rat

Appendix Table S2

Primer	Strand	Sequence (5' -3')
**qRT-PCR**		
B2M	F	TGC TCG CGC TAC TCT CTC TTT
	R	TCT GCT GGA TGA CGT GAG TAA AC
GAPDH	F	GGG AAA CTG TGG CGT GAT
	R	GGA GGA GTG GGT GTC GCT GTT
KAP1	F	AAG GAC CAT ACT GTG CGC TCT AC
	R	ACG TTG CAA TAG ACA GTA CGT TCA C
LIPA4_1	F	TCA CCA ATA TCC GCT GTT CTG
	R	GTC TGT TGG AGT TTA CTG GAG G
LIPA4_2	F	TGA TAC CCA GGC AAA CAG G
	R	TCT AAC AGT CAG GAC CCT CAG
HERVK14C_1	F	AAY AGC ACT GGA GCC CTT
	R	CGA CTG TGA TGG TTS AYT TTG
HERVK14C_2	F	GTA ATT GTG AGT ACC CAA AAT CTC
	R	ACC TTG TCC CAA TCT TTT AC
SVA D VNTR	F	GCT GCC CAT CGT CTG AG
	R	TCC TCA CCT CCC AGA CAG
ISG56	F	CCT GAA AGG CCA GAA TGA GG
	R	TCC ACC TTG TCC AGG TAA GT
CCL5	F	CCC AGC AGT CGT CTT TGT CA
	R	TCC CGA ACC CAT TTC TTC TCT
CXCL10	F	GTG GCA TTC AAG GAG TAC CTC
	R	GCC TTC GAT TCT GGA TTC AGA
Mxα	F	ATC CTG GGA TTT TGG GGC TT
	R	CCG CTT GTC GCT GGT GTC G
Ikβα	F	CTC CGA GAC TTT CGA GGA AAT
	R	GCC ATT GTA GTT GGT AGC CTT
**shRNA**		
shKAP1_1	F	GAT CCG TAA GCA CAG GTT TGG TCT CAG TTC AAG AGA CTG AGA CCA AAC CTG TGC TTA TTT TTT ACG CGT G
	R	AAT TCA CGC GTA AAA AAT AAG CAC AGG TTT GGT CTC AGT CTC TTG AAC TGA GAC CAA ACC TGT GCT TAC G
shKAP1_2	F	GAT CCG TAA GAA CTG GTA CTG GTG GTC TTC AAG AGA GAC CAC CAG TAC CAG TTC TTA TTT TTT ACG CGT G
	R	AAT TCA CGC GTA AAA AAT AAG AAC TGG TAC TGG TGG TCT CTC TTG AAG ACC ACC AGT ACC AGT TCT TAC G
**sgRNA**		
KAP1_ Ex1	F	CAC CGG AGC GCT TTT CGC CGC CAG
	R	AAA CCT GGC GGC GAA AAG CGC TCC
KAP1_Ex9	F	CAC CGC GTC CTG GCA CTA ACT CAA C
	R	AAA CGT TGA GTT AGT GCC AGG ACG C

STING		TCCATCCATCCCGTGTCCCAGGG
MAVS		CAGGGAACCGGGACACCCUC
Non-targeting sgRNA		ACGGAGGCTAAGCGTCGCAA
**DNA methylation**		
HERVK14C_LTR	F	AGG TTT AGG AAG GTG GAT TAT TTG
	R	ACA ACC CAA AAC TCT CAA ACT CTA C
OCT4	F	ATT TGT TTT TTG GGT AGT TAA AGG T
	R	CCA ACT ATC TTC ATC TTA ATA ACA TCC
SVAs	F	TTGTAATTTTTTTGTTTGATTTTTTTGT
	R	TACACTCCAACCTAAACACCATTAA
**Sequencing**		
T7	-	TAA TAC GAC TCA CTA TAG GG
CPPT_F	-	CAG GCC CGA AGG AAT AGA AG
**Reporter**		
Con_LTR	F	TCG AGG CAC GCG TTC TGG CGC CCA ACG TGG GGC TCC CCA TAA TCC
	R	TCG AGG ATT ATG GGG AGC CCC ACG TTG GGC GCC AGA ACG CGT GCC
Chr15_LTR	F	GCC TCG AGG GAT TAT GGG GAG CCC CAT GTT GGG CGC CAG AGC ACG CGT TGT GAG AAA GAG AGT TTC TGA GGT GC
	R	GCG GAT CCT GTT GGG GAA ACC AGC CC
LTR	F	GCC TCG AGA GAC CTG TGA GAA AGA GAG TTT CTG AGG TGC
	R	GCG GAT CCT GTT GGG GAA ACC AGC CC
**Taqman qPCR**		
GFP	F	CTG CTG CCC GAC AAC CAC
	R	ACC ATG TGA TCG CGC TTC TC
	Probe	CCA GTC CGC CCC TGA GCA AAG ACC
Human Albumin	F	GCT GTT CAT CTT GTG GGC TGT
	R	ACT CAT GGG AGC TGC TGG TTC
	Probe	[6FAM] CCT GTC ATG CCC ACA CAA ATC TCT CC [BHQ1]
**ChIP qPCR**		
ZNF180	F	TGATGCACAATAAGTCGAGCA
ZNF180	R	TGCAGTCAATGTGGGAAGTC
ZNF239	F	GGAGAAATCCCATGAGGGTAA
ZNF239	R	GGCTTTTGTGAGAATGTTTTCC
GAPDH	F	CACCGTCAAGGCTGAGAACG
GAPDH	R	ATACCCAAGGGAGCCACACC

Appendix Table S3

Gene Name	Species
2'-5'-oligoadenylate synthetase like(OASL)	Homo sapiens
TNF receptor superfamily member 21(TNFRSF21)	Homo sapiens
complement C1r subcomponent like(C1RL)	Homo sapiens
complement C1s(C1S)	Homo sapiens
complement C3(C3)	Homo sapiens
complement factor B(CFB)	Homo sapiens
complement factor D(CFD)	Homo sapiens
interferon gamma inducible protein 16(IFI16)	Homo sapiens
interferon induced protein with tetratricopeptide repeats 2(IFIT2)	Homo sapiens
major histocompatibility complex, class I, A(HLA-A)	Homo sapiens
major histocompatibility complex, class I, B(HLA-B)	Homo sapiens
proteasome subunit beta 9(PSMB9)	Homo sapiens

Appendix Figure S1 Legend.

Examples of KAP1-regulated repeat elements.

(A) Examples of ERV loci that are bound by KAP1 and potentially act as regulatory hubs through multiple transcription factor interactions as revealed by ENCODE data. (B) Repeats significantly downregulated (>2 fold where p=<0.05, using DESeq2) in KAP1 knockout HeLa clones compared to control cells based on mRNA-sequencing data. p = 0.0188 (HUERS-P1), 0.0228 (L1PBA1_5), 0.0417 (L1M2C_5). All numbers above bars depict fold changes compared to control cells (to one decimal place).

Appendix Figure S2 Legend.

Cytosine methylation acts on KAP1-regulated retrotransposons and prevents innate immune activation

(A) ERV reactivation (left) and partial ISG induction (right) in HeLa cells following depletion of KAP1 (day 6 time-point) with normalization to *B2M* and *GAPDH* respectively. This is a repeat experiment of that shown in Figure 4A. Two-tailed unpaired t tests were performed and p-values are: L1PA4: 0.009, 0.003; HERVK14C: 0.006, 0.004; SVA D VNTR: 0.012; CCL5: 0.005, 0.007. (B) PBMCs depleted for KAP1 (see Figure 1F and S1F) were assessed for induction of ISGs (day 6) by qRT-PCR with *GAPDH* normalization. Two-tailed unpaired *t* tests showed no significant difference: p values = 0.4083 for *ISG56* and p = 0.2401 for *CXCL10*. (C) 293T cells were treated with 5-AZA or a DMSO control and expression of endogenous repeats (left) and ISGs (right) was measured by qRT-PCR at day 6 post transduction with normalization to *B2M* and *GAPDH* respectively. p-values: HERVK14C: 0.018; SVA D VNTR: 0.005; CCL5: 0.004; CXCL10: 0.003; Mxa: 0.00006; IKba: 0.007. (D) HERVK14C LTR methylation over 4CpGs day 5 post shRNA or day 2 post 5-AZA treatment (see also Figure 4C). Mann-Whitney tests showed p values of 0.4280 (*shControl* vs. *shKAP1*) and 0.9634 (DMSO vs. 5-AZA). 5-AZA was added to all experiments at 7uM. (E) HeLa cell counts day 5 post transduction with *shKAP1* or *shControl* vectors. p values from unpaired t tests are 0.004 (experiment 1) and 0.0004 (experiment 2). All numbers above bars depict fold changes compared to control cells (to one decimal place).
