# Supplementary material for: KAP1 regulates endogenous retroviruses in adult human cells and contributes to innate immune control
Source: EMBO Rep. 2018 Jul 30;19(10):e45000. doi: 10.15252/embr.201745000 (PMC6172469; doi:10.15252/embr.201745000)
Supplement: Supplementary file 2 — Expanded View Figures PDF [file EMBR-19-e45000-s002.pdf]

Expanded View Figures

Figure EV1. Validation of cell types and functional validation of KAP1 knockout.

A Chromosome map showing the HERVK14C loci with at least one internal open reading frame and long terminal repeats. Note that many copies are present on the Y chromosome so are not relevant in female cells.

B NTERA-2 and 293T cells were stained for OCT4 intracellularly and were subjected to flow cytometry analysis. A PE isotype control antibody was used as a negative control.

C KAP1 knockout HeLa cells were validated using a known KAP1-KZFP target sequence [12,26]: Wild-type HeLa cells, KAP1 knockout cells, and KAP1 reconstituted knockout cells (+KAP1 cDNA) were doubly transduced with a PBS-Pro-GFP reporter vector (from [10]) and a vector expressing either the cognate ZFP, Zfp809, or a control ZFP, Zfp819 (left). KAP1 expression levels are shown by Western blot (right). In “Mock” controls, the ZFP expression vectors were replaced with same volume of media. Error bars show SD, $N = 3$. Two-tailed unpaired t-test P-values are as follows: HeLa wild type: 0.033; KAP1 KO clone_1: 0.3185; KAP1 KO clone_2: 0.0036. Note that P-values are not available for the KAP1 KO (bulk) bars only due to $N < 3$.

D KAP1 knockout 293T cells were validated using known KAP1-KZNF target sequences (constructs were a kind gift from David Haussler) [9]. KAP1 wild-type and knockout 293T cells were co-transfected with the luciferase reporter construct, a Renilla control plasmid and constructs expressing either ZNF91 or ZNF93 (see Materials and Methods). Two-tailed unpaired t-test P-values are as follows: WT SVA_ZNF91: < 0.0001; WT LINE1_ZNF93: < 0.0001; KO SVA_ZNF91: 0.6008; KO LINE1_ZNF93: 0.0841. Error bars show SD, $N = 3$.

E DNA methylation analysis of endogenous SVAs in 293T cells. Primers were designed on a SVA copy on chromosome 7 but primers recognize 219 copies of SVAs, some of which exhibit CpG deletions or mutations (shown by “x” on the CpG map). PCR for the *OCT4* gene body was used as a positive endogenous control for cytosine methylation.

F Shows an independent PBMC experiment with a different donor to that shown in Fig 1F with the same time point (day 6). Expression was normalized to *B2M*. Note that KD efficiency is measured on the bulk population of cells and not reflective of the KD efficiency within the population of cells most efficiently transduced, which is typically lower for PBMCs than cell lines. Two-tailed unpaired t-test P-values are as follows: <0.0001 (LIPA4) and (KAP1 normalized to GAPDH).

G, H Primary $CD4^+$ T cells were activated and transduced with KAP1 knockdown or control vectors. Cells were harvested at different time points for (G) Western blot and (H) qRT–PCR with *B2M* normalization. Two-tailed unpaired t-test P-values are as follows: 0.64 (HERVK14C_1; “shControl” compared to “Day 9 shKAP1.2”) and 0.04 (HERVK14C_2; “shControl” compared to “Day 9 shKAP1.2”). Error bars show SEM, $n = 2$.

I DNA methylation status of the HERVK14C LTR region on chromosome 15 in $CD4^+$ T cells as tested using bisulfite sequencing.

Data information: All numbers above bars depict fold changes compared to control cells (to one decimal place). ***$P < 0.001$, **$P < 0.01$, and *$P < 0.05$.
Source data are available online for this figure.

 © 2018 The Authors

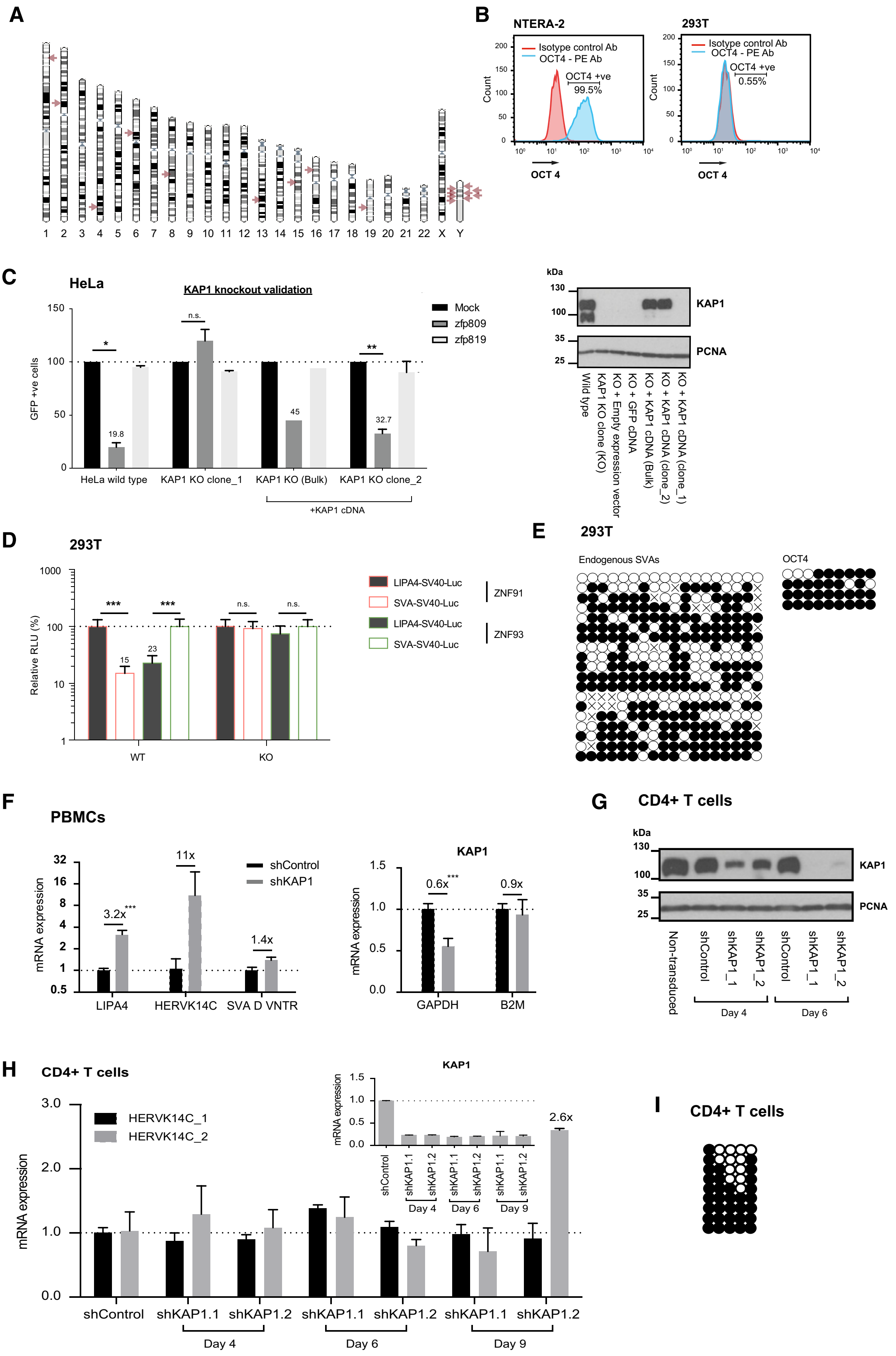
A
1
2
3
4
5
6
7
8
9
10
11
12
13
14
15
16
17
18
19
20
21
22
X
Y
B
NTERA-2
293T
Isotype control Ab
OCT4 - PE Ab
OCT4 +ve
99.5%
0.55%
Count
OCT 4
C
HeLa
KAP1 knockout validation
Mock
zfp809
zfp819
GFP +ve cells
19.8
45
32.7
n.s.
HeLa wild type
KAP1 KO clone_1
KAP1 KO (Bulk)
KAP1 KO clone_2
+KAP1 cDNA
kDa
KAP1
PCNA
Wild type
KAP1 KO clone (KO)
KO + Empty expression vector
KO + GFP cDNA
KO + KAP1 cDNA (Bulk)
KO + KAP1 cDNA (clone_2)
KO + KAP1 cDNA (clone_1)
D
293T
Relative RLU (%)
LIPA4-SV40-Luc
SVA-SV40-Luc
ZNF91
ZNF93
15
23
n.s.
WT
KO
E
293T
Endogenous SVAs
OCT4
F
PBMCs
mRNA expression
shControl
shKAP1
3.2x
11x
1.4x
LIPA4
HERVK14C
SVA D VNTR
KAP1
0.6x
0.9x
GAPDH
B2M
G
CD4+ T cells
kDa
KAP1
PCNA
Non-transduced
shControl
shKAP1_1
shKAP1_2
Day 4
Day 6
H
CD4+ T cells
HERVK14C_1
HERVK14C_2
mRNA expression
KAP1
2.6x
shControl
shKAP1.1
shKAP1.2
Day 4
Day 6
Day 9
I
CD4+ T cells

Figure EV1.

Figure EV2. Cellular genes are among the targets of KAP1 regulation.

A Examples of cellular genes upregulated (ZNF432, ZNF569, SNAI1, CYP17A1, CTGF shown) or downregulated (KAP1 shown) upon KAP1 knockout in HeLa cells based on mRNA-sequencing data. BigWig files were uploaded to the UCSC genome browser as URL links and the same scale (stated) applied to all samples.

B The top 100 most significantly downregulated genes (> 2-fold with a P_{adj}-value < 0.05) were converted to DAVID IDs and used for gene ontology analysis. All shown gene clusters were significantly enriched (P-values < 0.05) in the data set: signal peptide ($P = 8.9 \times 10^{-19}$), glycoprotein ($P = 3.6 \times 10^{-19}$), cell adhesion ($P = 2.5 \times 10^{-10}$), extracellular matrix ($P = 1.6 \times 10^{-8}$), EGF-like region ($P = 0.000004$), wounding response ($P = 0.000006$), plasma membrane ($P = 0.000029$), cell-cell adhesion ($P = 0.000160$), and innate immune response ($P = 0.000780$).

C Table showing that all upregulated KZNFs (from Fig 2C) are KAP1-bound according to ENCODE data.

D Venn diagrams showing that some KAP1 binding sites are common between human ESCs and 293T cells; 614 common binding sites were identified based on their presence in duplicate KAP1 ChIP-sequencing experiments in human ESCs as well as in duplicate KAP1 ChIP-sequencing experiments in 293T cells. Data are from [11] and ENCODE (293T cells).

 © 2018 The Authors

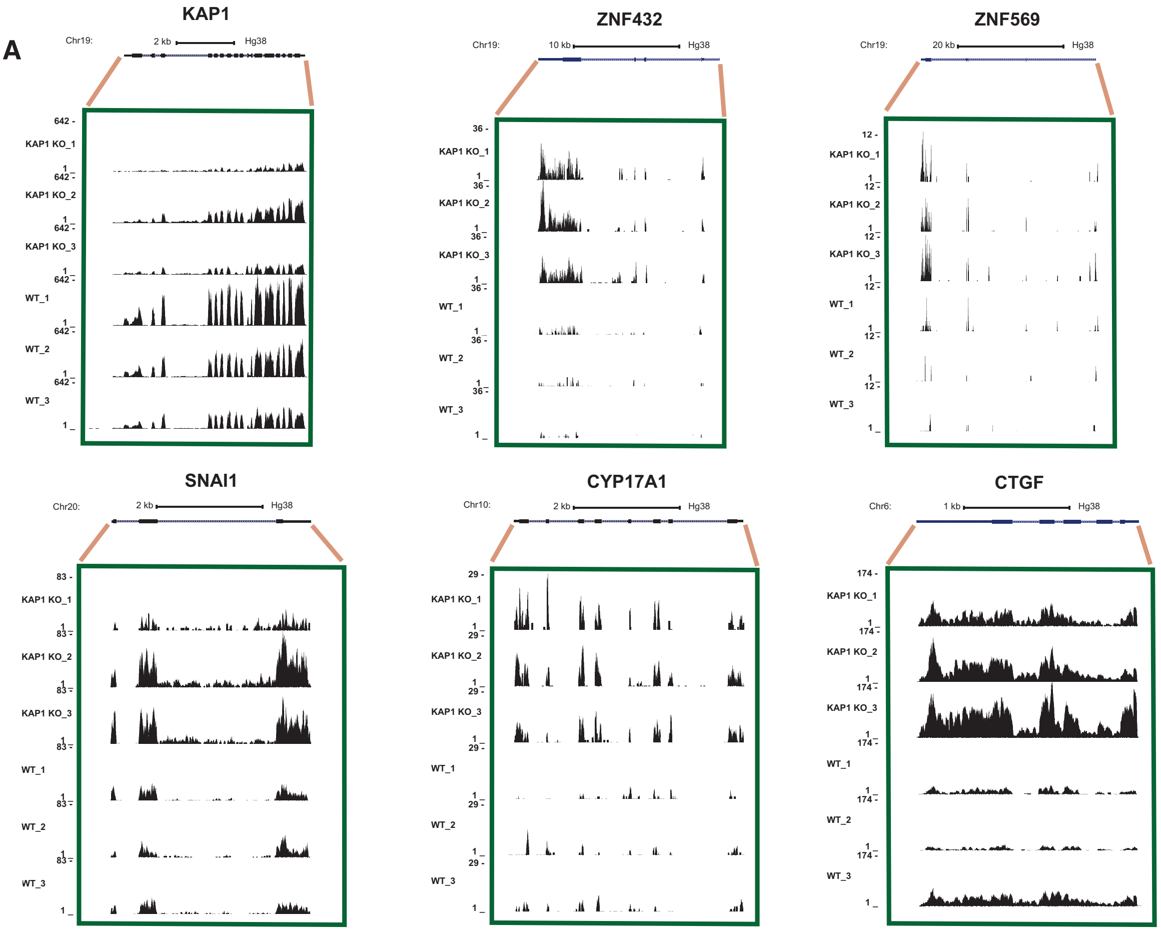

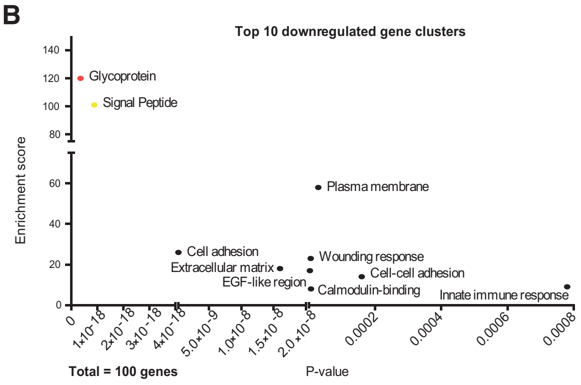

C

KZNF	Fold Up	H3K9me3 on 3′ end	H3K9me3 spreads to Promoter	KAP1 on 3′ end	KAP1 at Promoter
ZNF432	6.81	YES	YES	YES	YES
ZNF350	5.0	YES	YES	NO	YES
ZNF140	4.38	YES	YES	YES	YES
ZNF569	4.06	YES	YES	YES	YES
ZNF607	4.03	YES	YES	NO	YES
ZNF274	2.37	YES	YES	YES	YES
ZNF45	2.24	YES	NO	YES	NO
ZNF300	2.19	YES	YES	YES	YES
HKR1	2.15	YES	NO	YES	NO

Over 4-fold overexpressed
Over 2-fold overexpressed

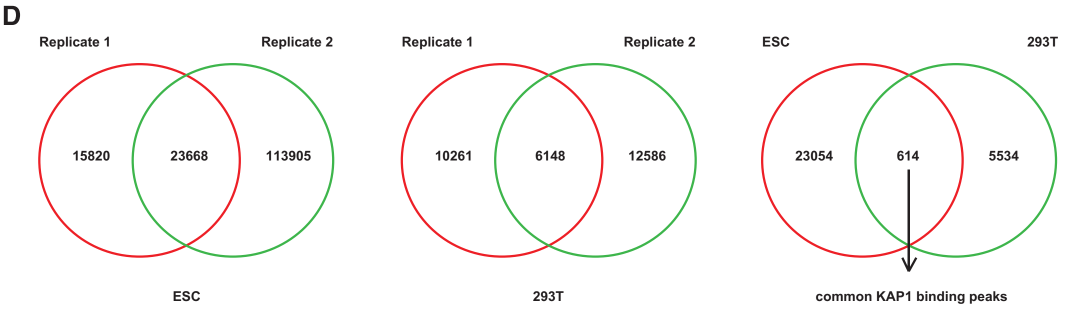

Figure EV2.

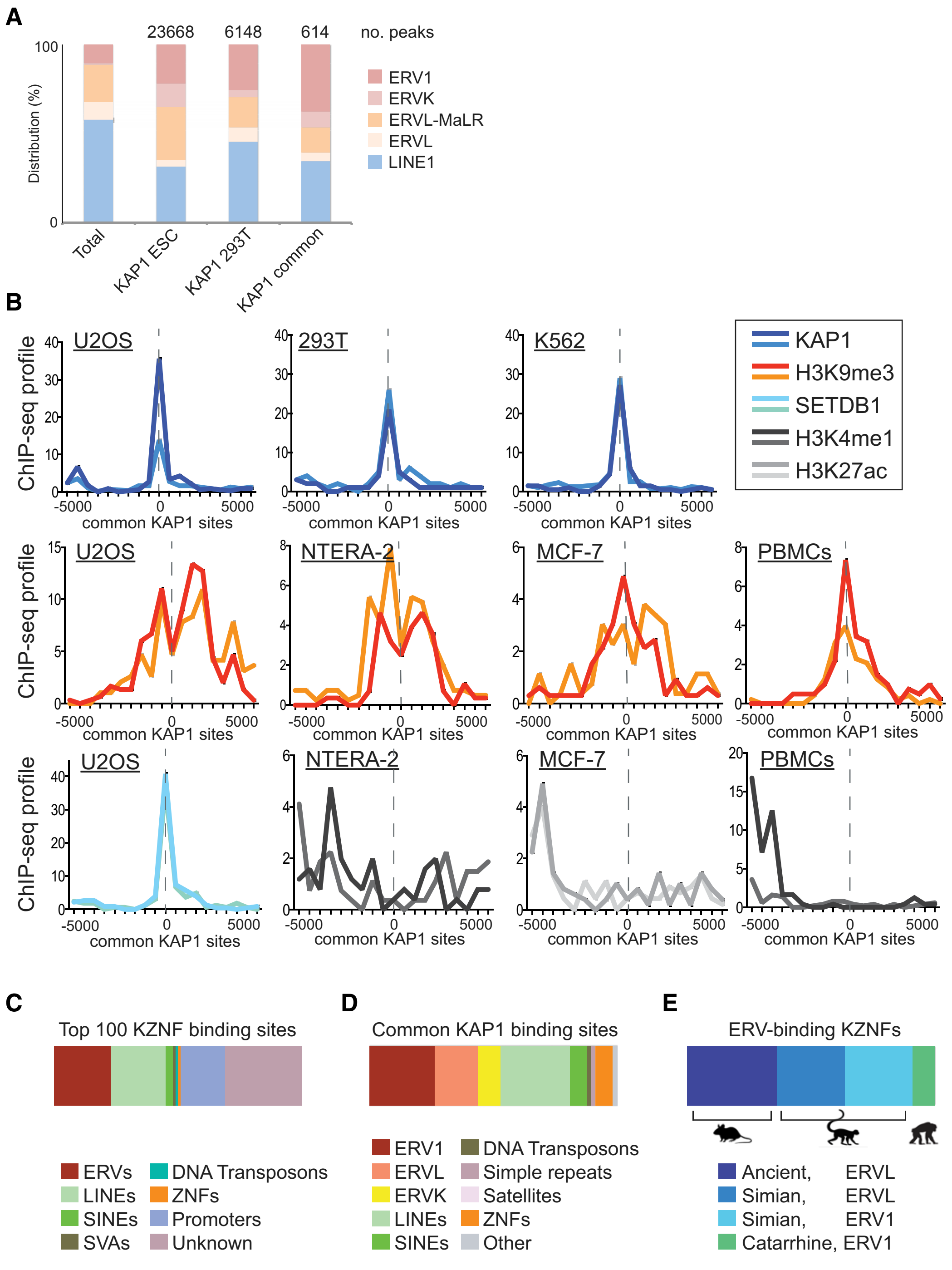

Figure EV3. Conserved KAP1 binding and H3K9me3 at ERVs and ZNFs.

A Comparison of the distribution of KAP1-bound ERVs and LINE1 elements between undifferentiated cells (human ESCs), differentiated cells (293Ts), and KAP1 common sites (sites conserved between human ESCs and 293T cells). Here, all genomic ERVs or LINE1s were intersected with the stated KAP1 peaks. Total number of intersections (15,399 for KAP1 ESC, 2,519 for KAP1 293T, and 742 for KAP1 common) is different to the total number of KAP1 peaks per dataset (shown) because some peaks intersect multiple or no repeats. For more information on the identity of each common KAP1 peak, see Dataset EV3.

B Complete data of which some is shown in Fig 2F: Genomic coordinates of the common KAP1 sites identified in Fig EV2D were subjected to ChIP-seq correlation analyses using ChIP-Cor software (see Materials and Methods). Each plot shows duplicate ChIP-seq experiments from ENCODE.

C The binding sites (where known [14,16]) within repeats and genes of the top 100 most highly expressed (defined by RNA-seq RPKM) KZNFs in 293T cells. These KZNFs were also confirmed to be expressed at the protein level (http://www.proteinatlas.org/cell).

D The binding sites within repeats and genes of KAP1 at common KAP1 sites.

E KZNFs within the top 100 most highly expressed group that are known to bind ERVs were assessed for their age and type of ERV that they bind to.

© 2018 The Authors

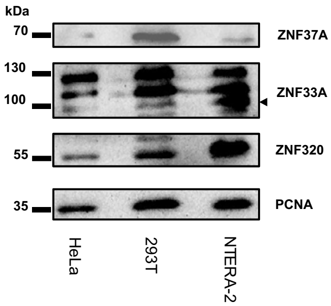

Figure EV4. KZNFs are expressed at the protein level in differentiated cells..

Western blots showing the protein expression levels of ZNF37A, ZNF33A, and ZNF320 in HeLa cells, 293T cells, and NTERA-2 cells. Arrowhead indicates expected size of ZNF33A.

Source data are available online for this figure.

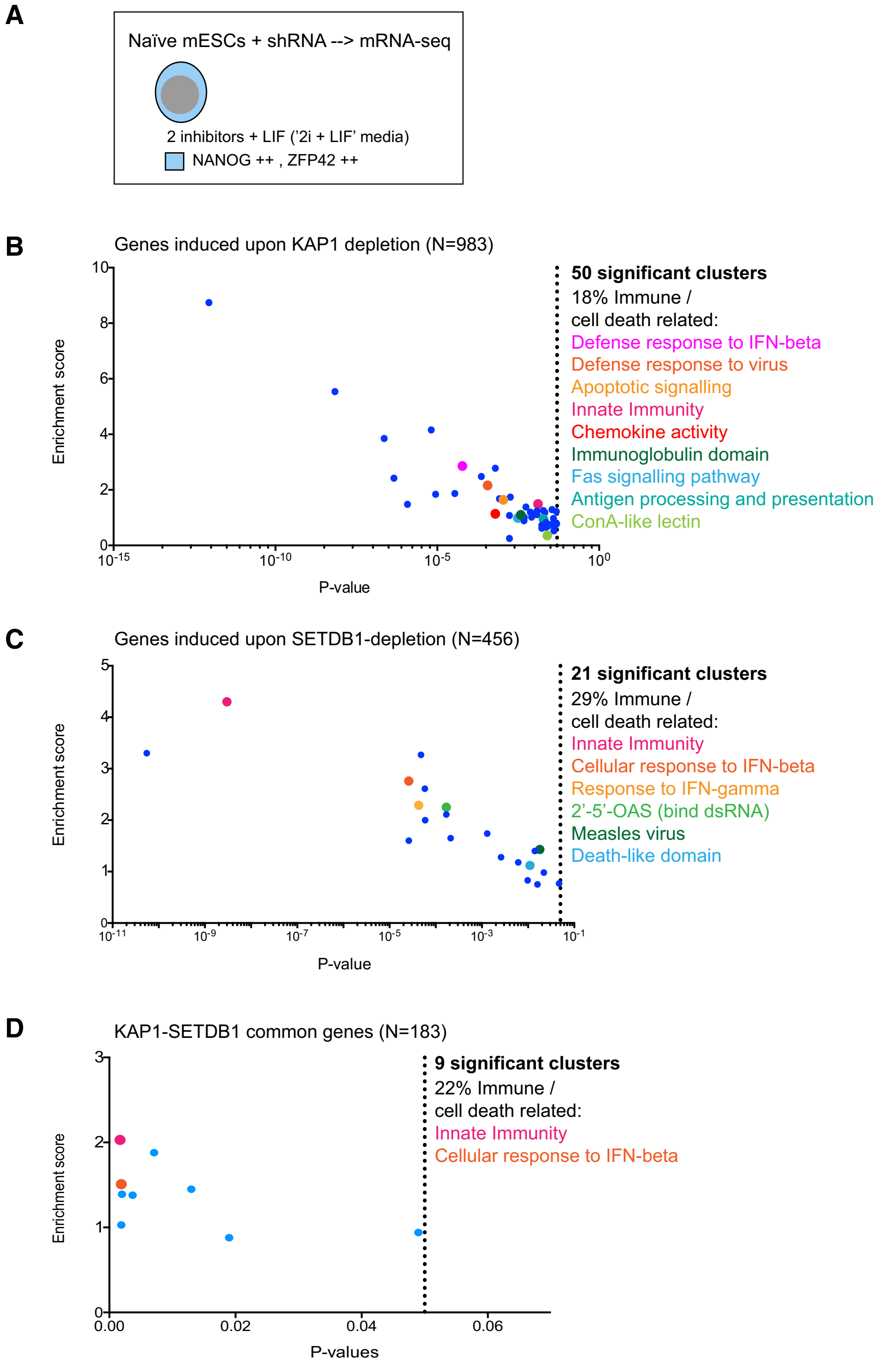

Figure EV5. Common genes induced upon KAP1 or SETDB1 depletion are most enriched in innate immunity.

- A We recently found that KAP1 and SETDB1 depletion exerts the greatest impact on retrotransposon transcriptional and protein upregulation in naïve mESCs [37]. Data from this work (GSE107840) were therefore subjected to DAVID gene ontology analyses to see whether innate immune genes were induced. We focused on genes induced > 2-fold with adjusted *P*-values < 0.05.
- B 983 genes were induced upon KAP1 depletion with 18% involved in immune or cell death-related clusters, which are listed and highlighted. Only clusters with *P*-values < 0.05 were included. Both innate and adaptive immune pathways were activated.
- C 456 genes were induced upon SETDB1 depletion with 29% involved in immune or cell death-related clusters as listed and highlighted. A clear innate immunity phenotype was activated.
- D 183 identical genes were induced in either KAP1- or SETDB1-depleted naive mESCs. Innate immunity was the most highly enriched gene cluster.

 © 2018 The Authors
