# Supplementary material for: KAP1 regulates endogenous retroviruses in adult human cells and contributes to innate immune control
Source: EMBO Rep. 2018 Jul 30;19(10):e45000. doi: 10.15252/embr.201745000 (PMC6172469; doi:10.15252/embr.201745000)
Supplement: Supplementary file 8 — Source Data for Expanded View [file EMBR-19-e45000-s010.zip › Source_Data_for_EV_Figures/Source_Data_for_FigureEV1.pdf]

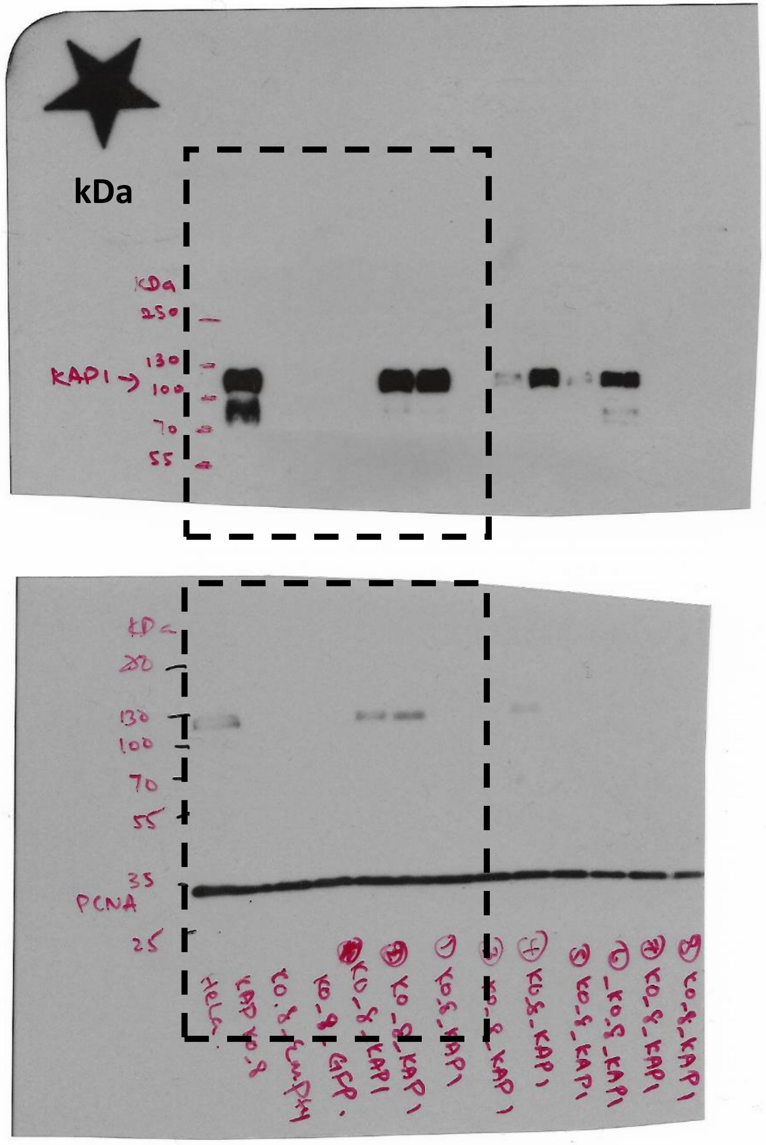


Sample order (Left to right):

1. HeLa Wild type
2. KAP1 KO clone
3. KAP1 KO + Empty expression vector
4. KAP1 KO + GFP cDNA
5. KAP1 KO + KAP1 (Bulk)
6. KAP1 KO + KAP1 cDNA (clone 2)
7. KAP1 KO + KAP1 cDNA (clone 1)

Figure EV1C

KAP1

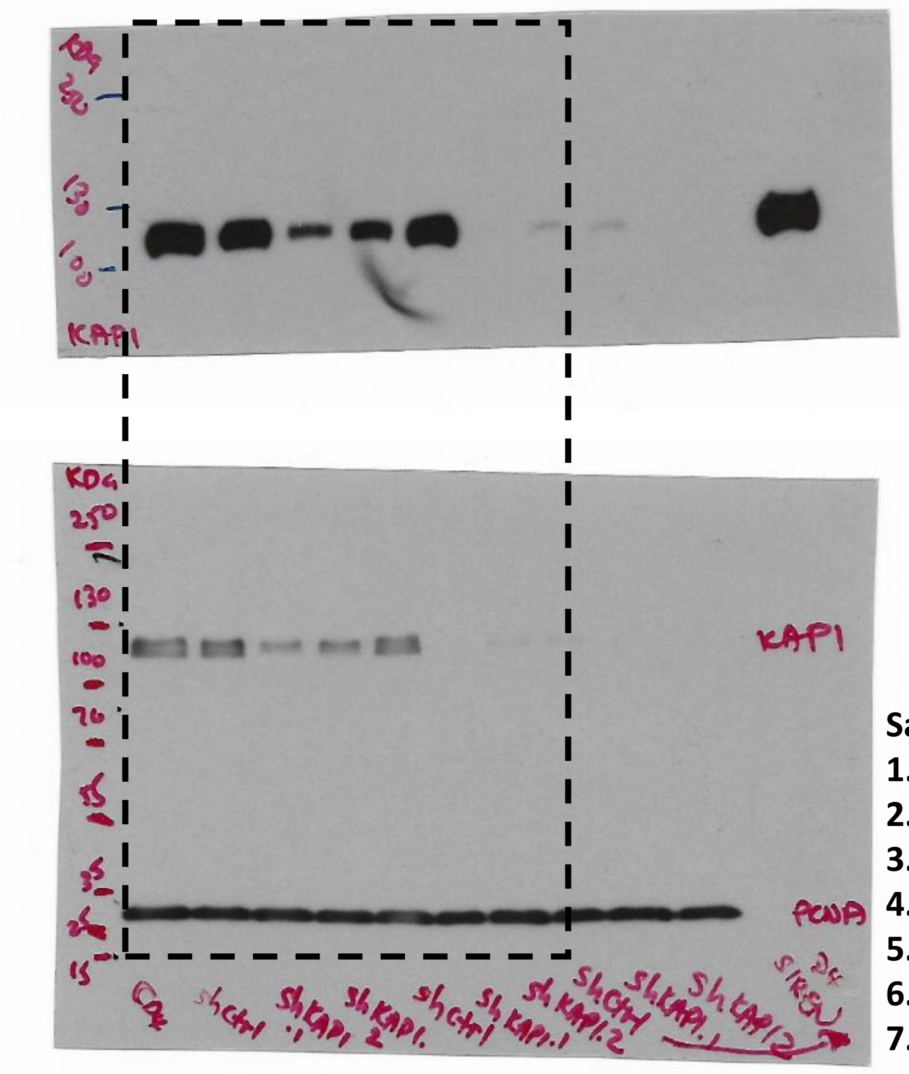


Sample order (Left to right):

1. CD4+ T cells Wild type
2. Day 4 shControl
3. Day 4 shKAP1_1
4. Day 4 shKAP1_2
5. Day 6 shControl
6. Day 6 shKAP1_1
7. Day 6 shKAP1_2

Figure EV1G
