# Supplementary figures and images for: KAP1 regulates endogenous retroviruses in adult human cells and contributes to innate immune control
Source: EMBO Rep. 2018 Jul 30;19(10):e45000. doi: 10.15252/embr.201745000 (PMC6172469; doi:10.15252/embr.201745000)

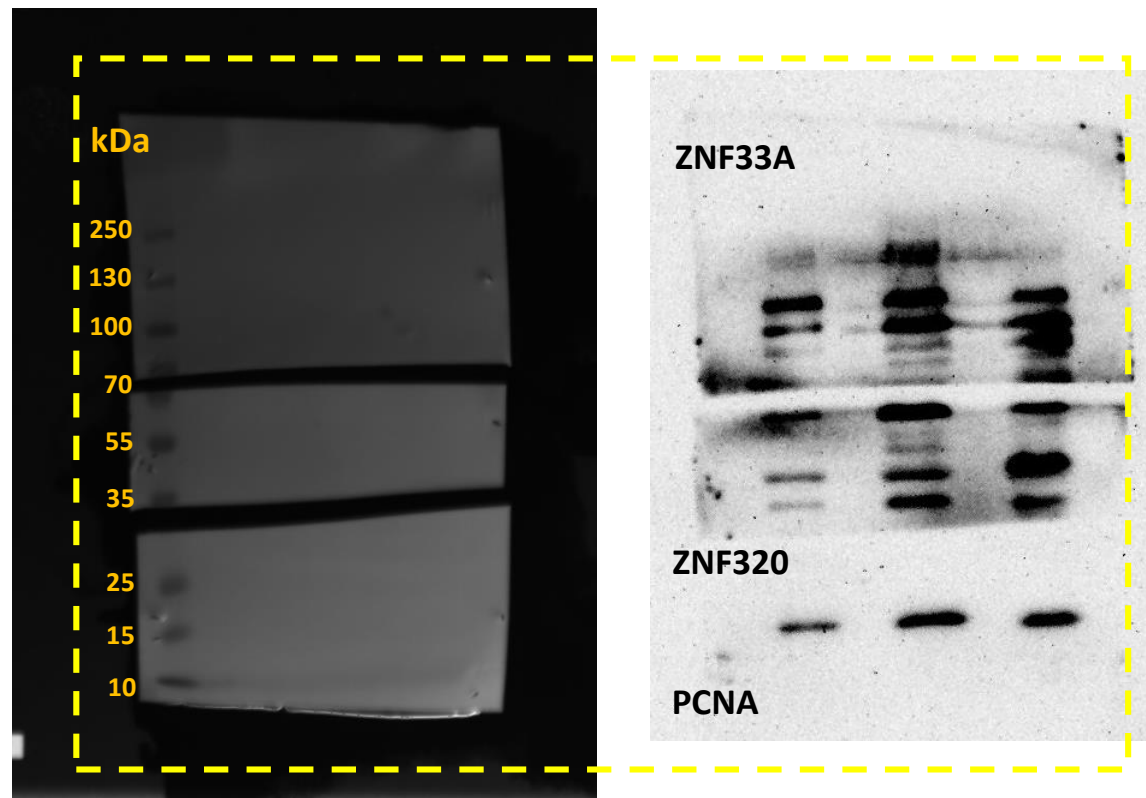

Sample order (Left to right):

1. HeLa
2. 293T
3. NTERA-2

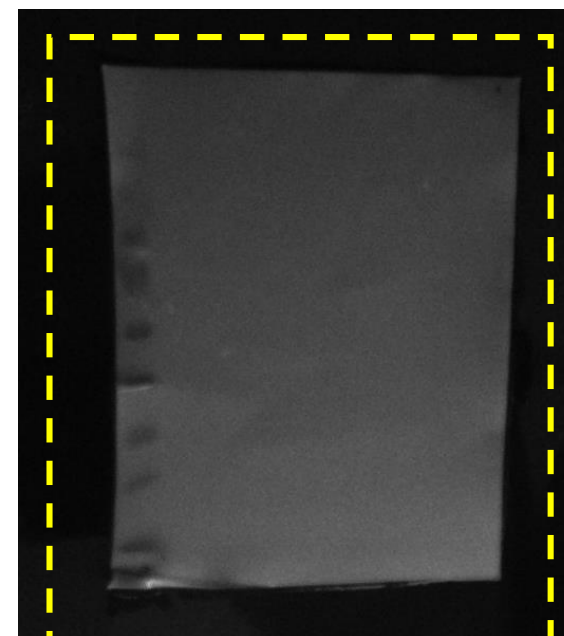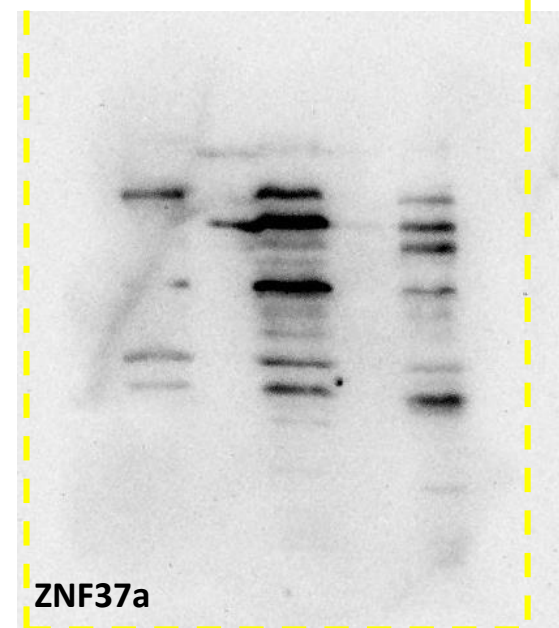

Figure EV4

Supplement: Supplementary file 8 — Source Data for Expanded View [file EMBR-19-e45000-s010.zip › Source_Data_for_EV_Figures/Source_Data_for_FigureEV4.pdf]

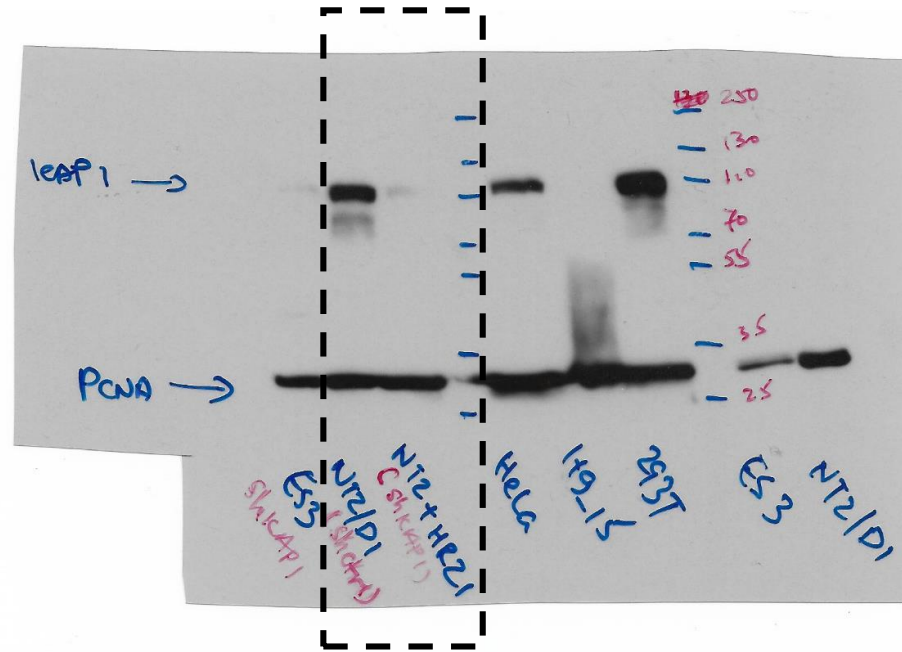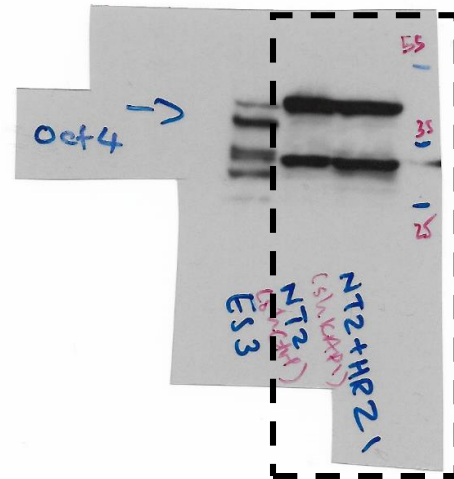

Figure 1C

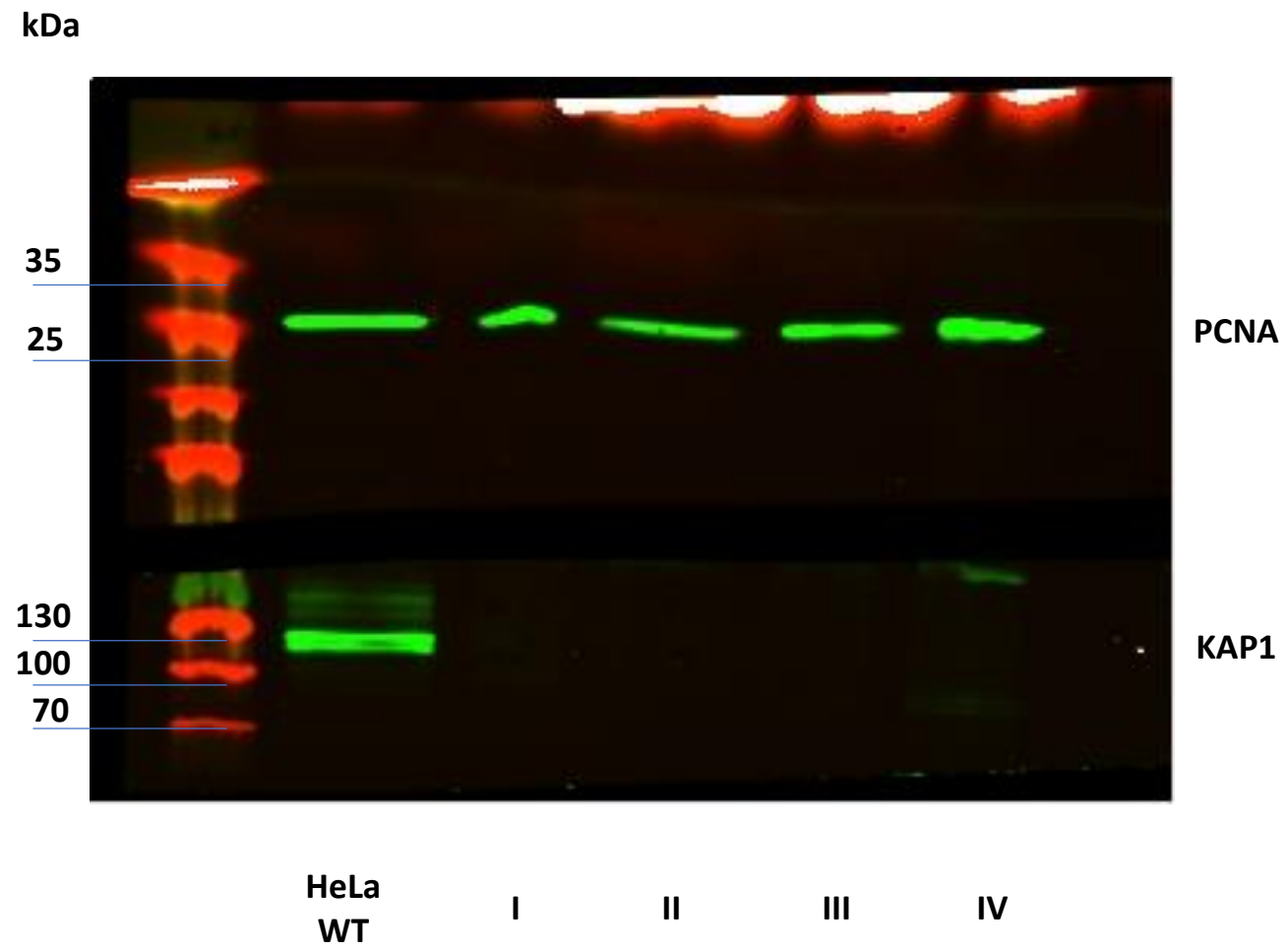

Figure 1D

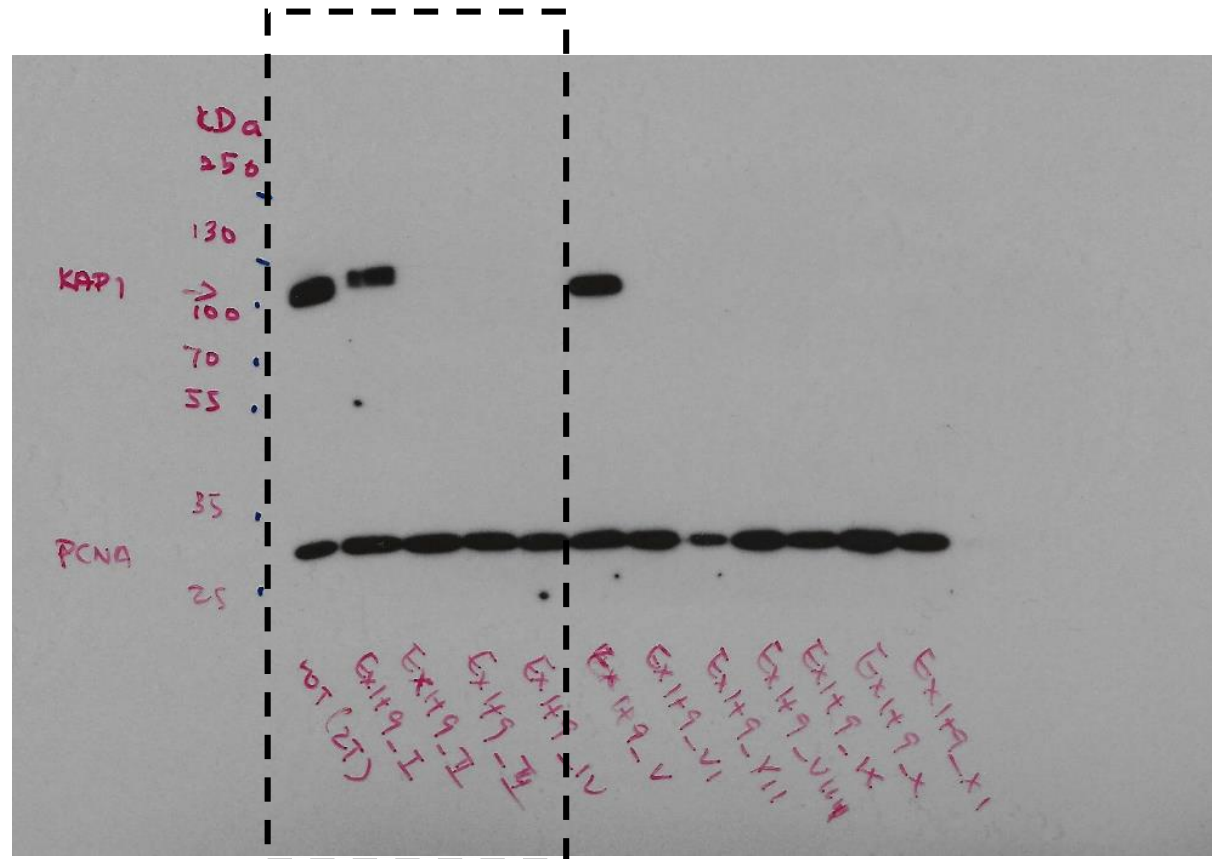

Figure 1E

Supplement: Supplementary file 10 — Source Data for Figure 1 [file EMBR-19-e45000-s008.pdf]

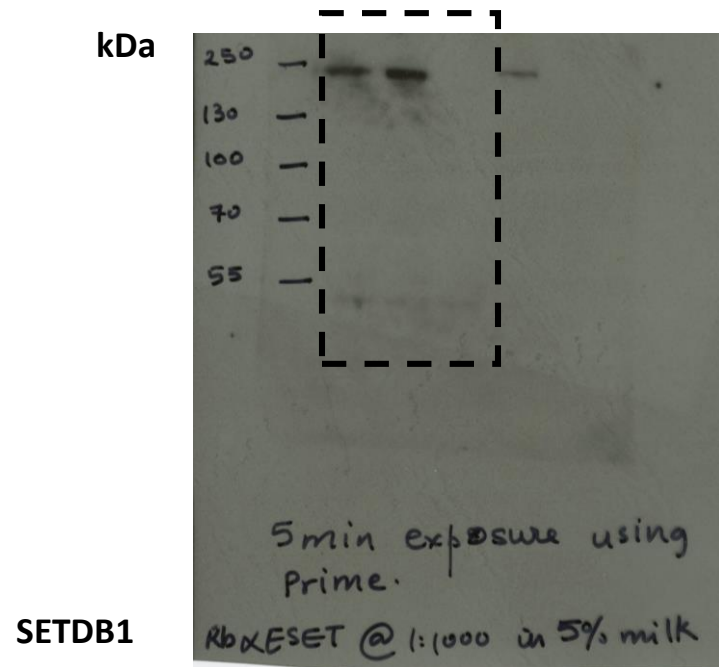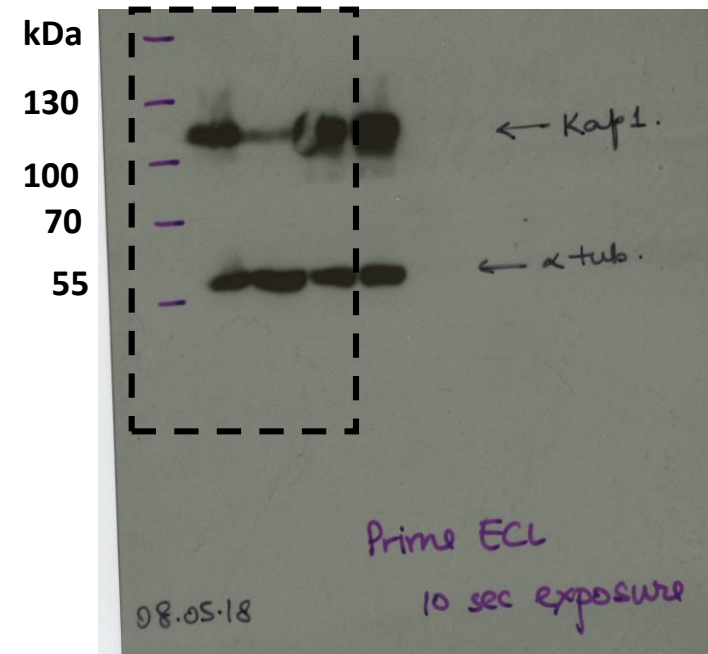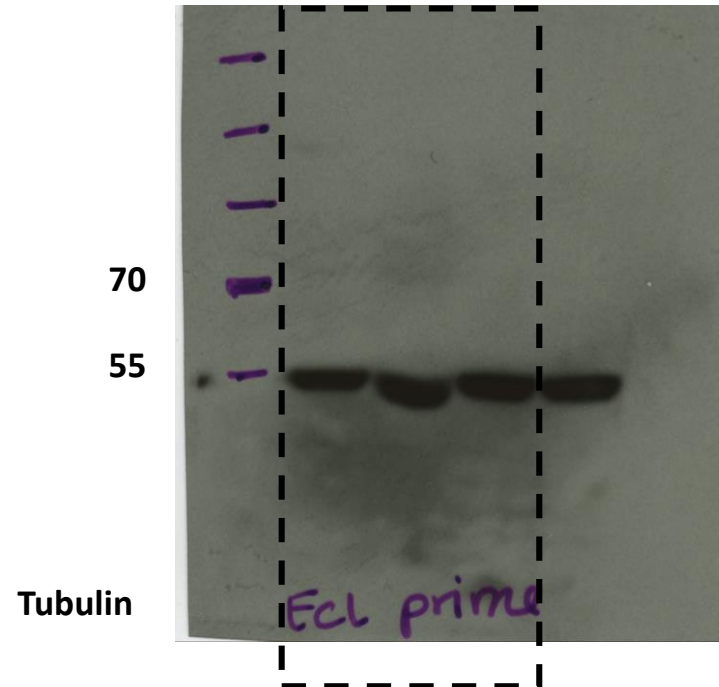

Sample order (Left to right):

1. shControl
2. shKAP1
3. shSETDB1

Figure 5B

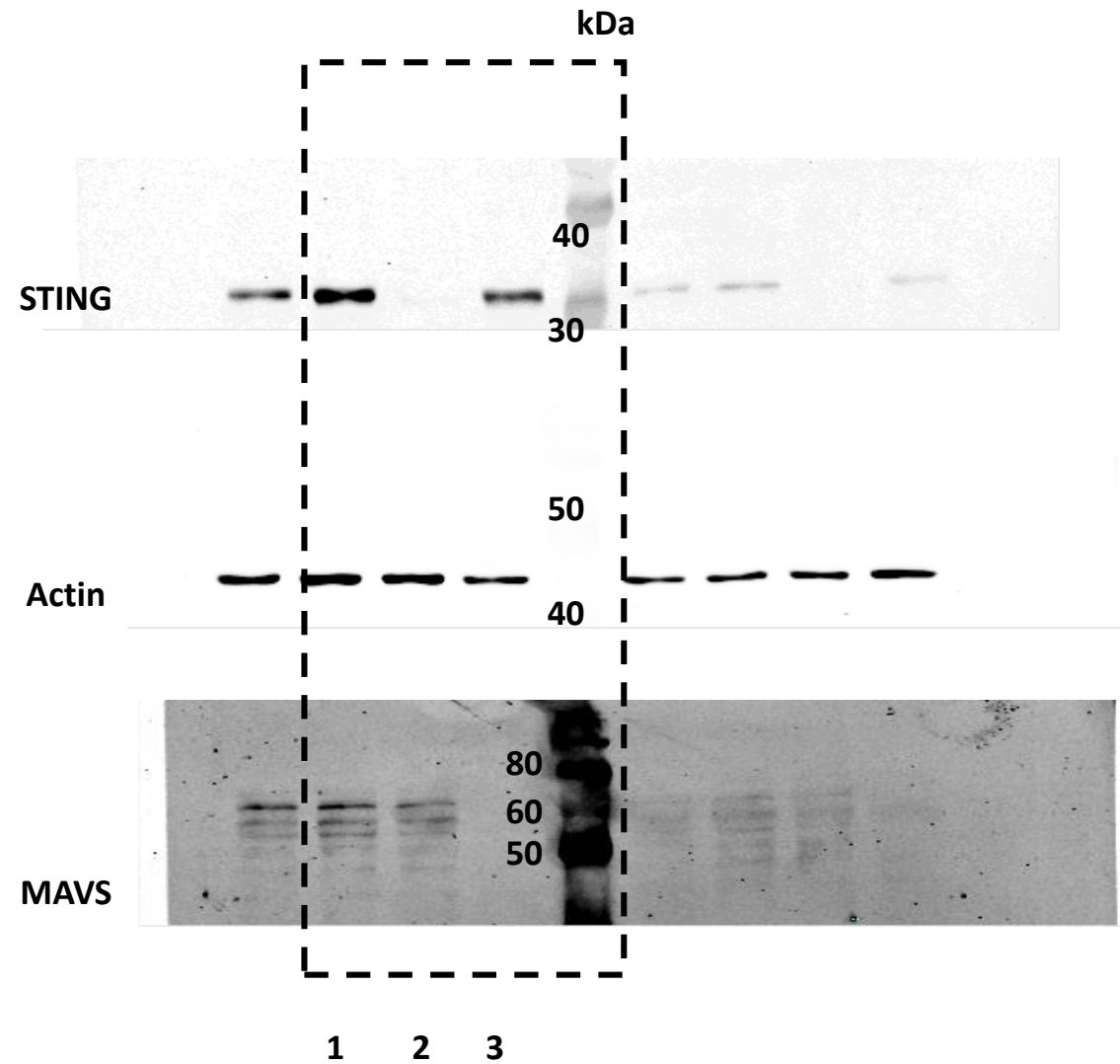

Supplement: Supplementary file 11 — Source Data for Figure 5 [file EMBR-19-e45000-s009.pdf]
